# Draft genome sequence of *Aurantiochytrium* spp. strains L3W, 9LR, 8W, 10W, and 10LW, a fatty acid-producing thraustochytrids from food wastes

**DOI:** 10.1128/mra.00653-24

**Published:** 2024-11-13

**Authors:** Toshikazu Suenaga, Yukine Mitarai, Tran Nhat Anh, Takehiko Gotoh, Wataru Nishijima, Satoshi Nakai

**Affiliations:** 1 Department of Chemical Engineering, Hiroshima University, Higashi-Hiroshima, Hiroshima, Japan; 2 Environmental Research and Management Center, Hiroshima University, Higashi-Hiroshima, Hiroshima, Japan; University of California Riverside, Riverside, California, USA

**Keywords:** *Aurantiochytrium*, thraustochytrid, draft genome sequence, Nanopore sequencing, Illumina sequencing

## Abstract

We report draft genome sequences of *Aurantiochytrium* spp. strains L3W, 9LR, 8W, 10W, and 10LW isolated from coastal environments. The strains were able to produce polyunsaturated fatty acids (PUFAs) such as docosahexaenoic acid (DHA) and eicosapentaenoic acid (EPA), confirming the metabolic pathways retained in the genomes.

## ANNOUNCEMENT

*Aurantiochytrium* sp. is a heterotrophic and halophilic thraustochytrid that produces polyunsaturated fatty acids (PUFAs) such as docosahexaenoic acid (DHA) and eicosapentaenoic acid (EPA). Because of the nature, the microorganism is industrially focused as an alternative source of PUFAs to fish oil (1). Previous studies demonstrate that *Aurantiochytrium* sp. strain L3W could grow on the food wastes and produced PUFAs (2, 3). Feeding the biomass mixture containing the biomass of strain L3W to the white leghorn hens as an eco-feed successfully enriched the DHA and EPA contents in eggs (4). To increase the efficiency of PUFA production or to discover applications for alternative carbon sources, metabolic studies based on genomic information are promising (5–9). Here, we report the genomes of five strains of *Aurantiochytrium* spp. that has been confirmed to produce PUFAs.

The strains 9LR, 8W, 10W, and 10LW were isolated from a mangrove leaf in Okinawa, Japan, using the same procedure as for strain L3W described in the previous study (10). The strains were incubated for 72 hours in 100 mL American Type Culture Collection 790By + medium with 1 mL of the antibiotics solution consisting of 10 g/L penicillin G potassium and 2.5 g/L streptomycin sulfate. Cultured cells were collected by centrifugation and washed twice with phosphate-buffered saline. Cells were lysed with 10% SDS buffer, and total nucleic acids were extracted by a phenol–chloroform method and subsequently purified with cetyltrimethylammonium bromide (11). The contained RNA was degraded with RNaseA (TaKaRa Bio, Inc., Japan), and the DNA was purified by ethanol precipitation (11). A paired-end DNA library was sequenced on a NovaSeq 6000 (Illumina, CA, USA). For long-read sequencing, DNA library was prepared by a ligation sequencing kit (SQK-LSK114; O.N.T., Oxford, UK) without a fragmentation procedure and sequenced on a MinION with Flongle (R10.4.1) adapter. The produced data were basecalled with Dorado (ver. 0.5.3) in the “duplex mode” and “super accurate model.” The poor-quality reads with low precision (< Q6) or too short (< 100 bp) were removed using NanoFilt (12), and then 40 bp from the beginning and 13 bp from the end were trimmed. The acquired short- and long-reads were assembled using MaSuRCA (ver. 4.1.0) (13), and finally scaffolds were generated. The positions of rRNA, tRNA, and protein-coding genes were predicted using Barrnap (ver. 0.9; specified eukaryote option) (14), tRNAscan-SE (ver. 2.0.9; search mode for eukaryotic tRNAs) (15), and BRAKER (ver. 3.0.7) (16), respectively. Completeness of the predicted genome was identified using BUSCO_v5 (17). Parameters used for the bioinformatics software were default values, unless otherwise noted. The information of the sequencer outputs, the constructed scaffolds, and the predicted genes is summarized in Table 1.

**TABLE 1:** Summary of draft genomes of *Aurantiochytrium* sp

Strain name	L3W	9LR	8W	10W	10LW
Genome size [Mbp]	62.34	63.64	61.75	63.01	59.89
GC content [%]	45.05	45.07	45.08	45.06	45.13
Number of scaffolds	65	58	109	46	186
N50 of assembly [Mbp]	2.002	1.932	1.216	1.929	0.5033
Number of rRNA	285	271	249	272	212
Number of tRNA	526	503	489	507	434
Num. of predicted genes	9,986	10,096	10,250	10,267	9,787
Output (Illumina) [Gbp]	28.16	11.04	19.94	19.76	12.88
Output (O.N.T.) [Gbp]^*a*^	1.573	0.9534	0.8339	0.9937	0.5165
Coverage (Illumina) [x]	451.8	173.5	322.9	313.6	215.1
Coverage (O.N.T.) [x]^*a*^	25.23	14.98	13.51	15.77	8.62
N50 of long reads [kbp]	9.131	27.72	12.02	26.56	17.43
BUSCO score (complete) [%]^*b*^	86.67	88.63	85.88	87.84	86.27
Acc. num. of the genome	BAAFGS000000000	BAAFGV000000000	BAAFGW000000000	BAAFGU000000000	BAAFGT000000000
Acc. num. of the genome assembly	GCA_041154985.1	GCA_041155045.1	GCA_041155065.1	GCA_041155025.1	GCA_041155005.1
Acc. num. of BioSample	SAMD00787331	SAMD00787334	SAMD00787335	SAMD00787333	SAMD00787332

^*a*^ O.N.T. stands for Oxford Nanopore Technologies Ltd.

^*b*^ The program version was BUSCO_v5, and the selected ortholog set was “Eukaryota.”

The genome sizes were estimated to be 59.89 to 63.64 Mbp, which were the same order of magnitude as the related species previously reported (5). Based on the 18S rRNA sequences, these strains are phylogenetically close to *A. limacinum* or *A. acetophilum*. The genomes reported in this study are promised to contribute to the elucidation of metabolism of PUFA production and industrial application of *Aurantiochytrium* sp.

## Data Availability

The sequencing data have been deposited at DDBJ as the BioProject (PRJDB18130) and BioSample (SAMD00787331-SAMD00787335). The accession numbers of genome and assembly for each strain are listed in Table 1.

## References

[1] Lemahieu C, Bruneel C, Dejonghe C, Buyse J, Foubert I. 2016. The cell wall of autotrophic microalgae influences the enrichment of long chain omega-3 fatty acids in the egg. Algal Res 16:209–215. doi:10.1016/j.algal.2016.03.015

[2] Suenaga T, Nakai S, Umehara A, Nishijima W, Gotoh T, Humaidah N. 2023. Valorization of solid food waste as a source of polyunsaturated fatty acids using Aurantiochytrium sp. L3W. Waste Biomass Valor 14:2945–2956. doi:10.1007/s12649-023-02072-0

[3] Pamungkas B, Asao W, Nakai S, Nii T, Nishijima W, Gotoh T, Suenaga T, Humaidah N, Tsudzuki M. 2024. Application of eco-feed produced by Aurantiochytrium sp. L3W using solid food waste for producing Hirodai-dori products containing omega-3 fatty acids. J Chem Eng Jpn 57:2333037. doi:10.1080/00219592.2024.2333037

[4] Asao W, Nakai S, Nii T, Nishijima W, Gotoh T, Humaidah N. 2023. Application of an eco-feed produced by culturing Aurantiochytrium sp. strain L3W using solid food waste to produce poultry products enriched with ω-3 fatty acids. J Mater Cycles Waste Manag 25:3346–3354. doi:10.1007/s10163-023-01757-x

[5] Zhu X, Li S, Liu L, Li S, Luo Y, Lv C, Wang B, Cheng CHK, Chen H, Yang X. 2020. Genome sequencing and analysis of Thraustochytriidae sp. SZU445 provides novel insights into the polyunsaturated fatty acid biosynthesis pathway. Mar Drugs 18:118. doi:10.3390/md18020118 32085426 PMC7073664

[6] Seddiki K, Godart F, Aiese Cigliano R, Sanseverino W, Barakat M, Ortet P, Rébeillé F, Maréchal E, Cagnac O, Amato A. 2018. Sequencing, de novo assembly, and annotation of the complete genome of a new thraustochytrid species, strain CCAP_4062/3. Genome Announc 6:e01335-17. doi:10.1128/genomeA.01335-17 29545303 PMC5854782

[7] Song Z, Stajich JE, Xie Y, Liu X, He Y, Chen J, Hicks GR, Wang G. 2018. Comparative analysis reveals unexpected genome features of newly isolated thraustochytrids strains: on ecological function and PUFAs biosynthesis. BMC Genomics 19:541. doi:10.1186/s12864-018-4904-6 30016947 PMC6050695

[8] Liu B, Ertesvåg H, Aasen IM, Vadstein O, Brautaset T, Heggeset TMB. 2016. Draft genome sequence of the docosahexaenoic acid producing thraustochytrid Aurantiochytrium sp. T66. Genom Data 8:115–116. doi:10.1016/j.gdata.2016.04.013 27222814 PMC4873566

[9] Ji X-J, Mo K-Q, Ren L-J, Li G-L, Huang J-Z, Huang H. 2015. Genome sequence of Schizochytrium sp. CCTCC M209059, an effective producer of docosahexaenoic acid-rich lipids. Genome Announc 3:00819–15. doi:10.1128/genomeA.00819-15 PMC4541280 26251485

[10] Nakai S, Das A, Maeda Y, Humaidah N, Ohno M, Nishijima W, Gotoh T, Okuda T. 2021. A novel strain of Aurantiochytrium sp. strain L3W and its characteristics of biomass and lipid production including valuable fatty acids. J Water Environ Technol 19:24–34. doi:10.2965/jwet.20-087

[11] Suenaga T, Aoyagi T, Hosomi M, Hori T, Terada A. 2018. Draft genome sequence of Azospira sp. strain I13, a nitrous oxide-reducing bacterium harboring clade II type nosZ. Genome Announc 6:e00414-18. doi:10.1128/genomeA.00414-18 29773628 PMC5958258

[12] De Coster W, D’Hert S, Schultz DT, Cruts M, Van Broeckhoven C. 2018. NanoPack: visualizing and processing long-read sequencing data. Bioinformatics 34:2666–2669. doi:10.1093/bioinformatics/bty149 29547981 PMC6061794

[13] Zimin AV, Marçais G, Puiu D, Roberts M, Salzberg SL, Yorke JA. 2013. The MaSuRCA genome assembler. Bioinformatics 29:2669–2677. doi:10.1093/bioinformatics/btt476 23990416 PMC3799473

[14] Seemann T. 2024. barrnap. https://github.com/tseemann/barrnap.

[15] Lowe TM, Eddy SR. 1997. tRNAscan-SE: a program for improved detection of transfer RNA genes in genomic sequence. Nucleic Acids Res 25:955–964. doi:10.1093/nar/25.5.955 9023104 PMC146525

[16] Hoff KJ, Lomsadze A, Borodovsky M, Stanke M. 2019. Whole-genome annotation with BRAKER. Methods Mol Biol 1962:65–95. doi:10.1007/978-1-4939-9173-0_5 31020555 PMC6635606

[17] Manni M, Berkeley MR, Seppey M, Zdobnov EM. 2021. BUSCO: assessing genomic data quality and beyond. Curr Protoc 1:e323. doi:10.1002/cpz1.323 34936221

